# 11.N. Workshop: School-based sexual health education: from evidence to implementation and evaluation

**DOI:** 10.1093/eurpub/ckac129.739

**Published:** 2022-10-25

**Authors:** Organised by: University of Pisa (Italy), Federal Centre for Health Education - WHO Collaborating Centre for Sexual and Reproductive Health, EUPHA-IDC, Chair persons: Lara Tavoschi (Italy), Johanna Marquardt (Germany)

## Abstract

Sexual and reproductive health (SRH) is a key dimension of health and well-being of individuals. Sexuality Education (SE) is one of the most important means of promoting sexual well-being of youth and a key component of HIV and sexually transmitted infections (STIs) prevention. International treaties and global health organisations explicitly urge governments to take the necessary measures to ensure the provision of SE, that should: be age-appropriate, start as soon as possible, promote mature decision-making, be directed towards improving gender inequalities and engage young people in all phases of activities. It is widely recognised that school plays a central role in learning about health and health promotion skills, including sexuality and sexual health. Although school-based SE (SBSE) alone may not be enough to ensure the rights of young people to SRH and prevent STIs, school programs are a very cost-effective way to contribute to these goals. Moreover, addressing these topics in schools is a thematic indicator for monitoring the UN Education Agenda 2030. Existing evidence indicates a great variability in the offer of school-based SE throughout the world. GEM report (2016) reveals that basic school curricula and educational standards rarely include SE programs, and multi-country reviews show limited progress in developing national strategies for the implementation of SE or in developing high-quality programs implemented on a large scale. Theoretical and research evidence indicate that SE, and in particular Comprehensive Sexuality Education (CSE), positively impacts the behaviour of young people, improving SRH outcomes by reducing risk-taking. UNESCO in 2018 defined CSE as “a curriculum-based process of teaching and learning about the cognitive, emotional, physical and social aspects of sexuality. It aims to equip children and young people with knowledge, skills, attitudes and values that will empower them to: realize their health, well-being and dignity; develop respectful social and sexual relationships; consider how their choices affect their own well-being and that of others; and, understand and ensure the protection of their rights throughout their lives”. In 2022, Italy is one of a few European countries that still lacks a comprehensive approach to SE that is coherently and equally implemented across the country and SE is not yet included in Italian schools’ curricula. As a consequence, several studies conducted on Italian young people report that they have poor sexual health knowledge, a low and inconsistent use of condoms and contraception, very little access to SRH services such as youth sexual health clinics and high levels of gender-based violence and homotransphobia. Moreover, 20% of all STIs detected in Italy affect young people. Yet, according to Italian students, school has clearly been pinpointed as the most appropriate place to receive information about SE.

**Key messages:**

• Some European countries, including Italy, do not include SE in national school curricula. Often, available SBSE programs lack a comprehensive approach, with limited coverage across the countries.

• Including CSE in national school curricula is an action urgently needed in order to provide young people with evidence-based, age-appropriate and accurate information on SRH and wellbeing.

